# *In vitro* flow cytometry assay to assess primary human and mouse macrophage phagocytosis of live cells

**DOI:** 10.1016/j.xpro.2023.102240

**Published:** 2023-04-18

**Authors:** Samantha Y. Liu, Naomi Mulugeta, Stephanie K. Dougan, Li Qiang

**Affiliations:** 1 Dana-Farber Cancer Institute, Boston, MA 02215, USA; 2 Harvard College, Cambridge, MA 02138, USA; 3 Harvard Medical School, Boston, MA 02215, USA

**Keywords:** Cell Biology, Flow Cytometry/Mass Cytometry, Cancer, Immunology

## Abstract

Although tumor-associated macrophages are generally immunosuppressive, macrophages may also promote tumor clearance via phagocytosis of live tumor cells. Here, we present a protocol for assessing macrophage engulfment of tumor cells *in vitro* using flow cytometry. We describe steps for cell preparation, reseeding macrophages, and setting up phagocytosis. We then detail procedures for collecting samples, staining macrophages, and flow cytometry. The protocol is applicable to both mouse bone-marrow-derived macrophages and human monocyte-derived macrophages.

For complete details on the use and execution of this protocol, please refer to Roehle et al. (2021).^1^

## Highlights

• Phagocytosis of live tumor cells by primary mouse or human macrophages
• Streamlined protocol for *in vitro* differentiation of human macrophages from PBMCs
• Analysis of phagocytosis in adherent macrophages by flow cytometry
• Use of cIAP1/2 antagonism and cytokines to induce phagocytosis

## Abstract (editor highlights)

Publisher’s note: Undertaking any experimental protocol requires adherence to local institutional guidelines for laboratory safety and ethics.

## Graphical abstract

**Figure:**
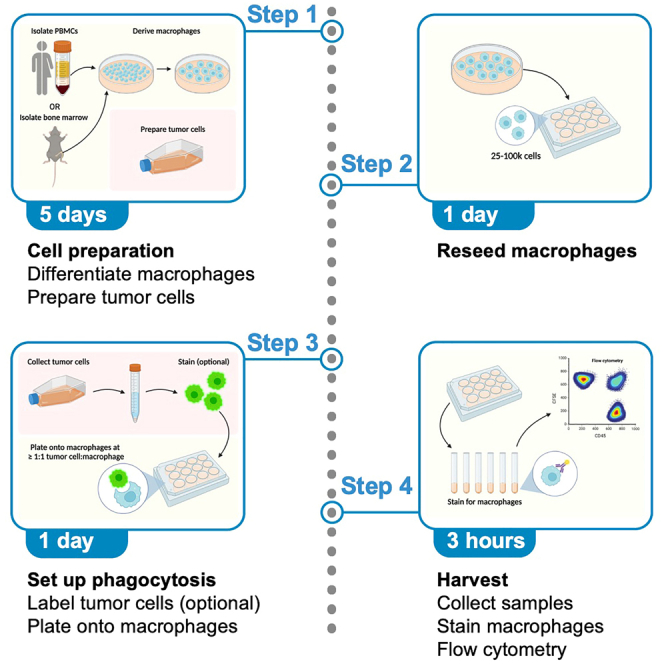

## Before you begin

The protocol below describes the specific steps for assessing primary human and mouse macrophage phagocytosis of live cells.

### Institutional permissions

These studies were conducted in accordance with the Declaration of Helsinki and the Belmont Report. Anonymous healthy donor leukopacks were obtained from the Kraft Family Blood Donor Center at Dana-Farber Cancer Institute and Brigham and Women’s Hospital, protocol T0363. Institutional permission must be obtained prior to working with human blood.

Mice were housed at the Dana-Farber Cancer Institute and were maintained according to protocols approved by the DFCI Institutional Animal Care and Use Committee (IACUC) (#14-019 and #14-037). Institutional permission must be obtained prior to working with live mice.

### Preparation of macrophages

**Timing: 5 days**

1. Differentiation of mouse macrophages:
  a. Isolate mouse bone marrow.
    i. Euthanize mouse by CO_2_ asphyxiation or other approved method as specified by institutional guidelines for humane euthanasia.
    ii. Spray mouse abdomen and hind legs with 70% ethanol.
    iii. Open the abdomen using sterile scissors and remove the skin from each hind leg.
    iv. Cut each hind leg above the hip joint and below the knee joint to isolate the femur with adjacent joints intact. Be careful not to break the femurs.
    v. Using scissors, forceps, and Kimwipes, gently clean off the muscles and tissues from the bones.
    vi. Rinse the bones once in 70% ethanol and then twice in PBS.
    vii. Once all bones are collected and rinsed, move to a sterile biosafety cabinet.
    viii. Prepare a 10-mL syringe with a 27-gauge needle and fill the syringe with ice-cold PBS.
    ix. Cut off the ends of each bone using sterile scissors. Using the syringe, flush out the bone marrow into a sterile dish. Continue flushing until the bone turns translucent, using more PBS as needed.
    x. Once all bone marrow is collected, gently pipet the mixture up and down with a 10 mL serological pipette, then filter the mixture through a 40 μM filter into a 50 mL conical tube.
    xi. Centrifuge at 400 × *g* for 5 min at 4°C to pellet cells and aspirate the supernatant.
  b. Lyse red blood cells.
    i. Resuspend the cells in 1 mL of ACK lysis buffer, leave for 30 s, then quench with 9 mL PBS. Remove a small aliquot for counting.
    ii. Centrifuge at 400 × *g* for 5 min at 4°C.
    iii. Count cells using a hemocytometer.
    iv. Aspirate supernatant.
  c. Plate cells.
    i. Resuspend cells in RPMI complete (see materials and equipment) supplemented with 20 ng/mL of recombinant murine M-CSF. Aim for an optimal concentration of 500,000 cells/mL. For differentiation in 150 × 15 mm petri dishes, plate 10–15 million cells in 20–30 mL media per plate.
    ii. Plate cells onto petri dishes and incubate at 37°C and 5% CO_2_ for five days.
  d. Feed cells on day 2 and day 4 of culture.
    i. For each petri dish, add the same volume of complete RPMI with 20 ng/mL of M-CSF as originally plated. If the plate is too full, aspirate half of the previous media. Otherwise, add fresh media to the existing volume.
2. Differentiation of human macrophages:
  a. Obtain a leukapheresis collar from a healthy human donor according to institutional protocols.
  b. Isolate peripheral blood mononuclear cells (PBMCs).
    i. In a sterile hood, spray collar with 70% ethanol and dry off with a Kimwipe.
    ii. Cut both sides of the collar with sterile scissors and let the blood drip into a 50 mL conical tube. Approximately 10 mL blood can be obtained from one collar.
    iii. Mix blood with equal volume PBS.
    iv. In a second 50 mL conical tube, prepare a volume of Ficoll-Paque that is equal to the original blood volume.
    v. Gently and slowly, pipet the blood/PBS mixture on top of the Ficoll-Paque. The two layers should be distinctly separated. Take care not to agitate the tube or use too much force when pipetting.
    vi. Centrifuge at 400 × *g* for 30 min at 4°C with SLOW acceleration and NO BRAKE settings.
    vii. Carefully isolate the white buffy coat at the interface between the serum and Ficoll layer. Wash in 50 mL of PBS.
    viii. Centrifuge at 400 × *g* for 5 min at 4°C.
  c. Plate cells.
    i. Count cells using a hemocytometer.
    ii. Resuspend cells in RPMI complete supplemented with 50 ng/mL of recombinant human M-CSF. For differentiation in 150 × 15 mm petri dishes, plate 10–15 million cells in 20–30 mL media per plate.
    iii. Plate 1–2 million cells per mL onto petri dishes and incubate at 37°C and 5% CO_2_ for five days.
  d. Feed cells on day 2 and day 4 of culture.
    i. For each petri dish, add the same volume of RPMI complete with 50 ng/mL of M-CSF as originally plated. If the plate is too full, aspirate half of the previous media. Otherwise, add fresh media to the existing volume.

### Maintenance of target tumor cells

**Timing: 5 days**

3. Culture target tumor cells:
  a. Thaw a cryogenic vial of the tumor cells briefly in a 37°C water bath.
  b. Mix the thawed cells with 9 mL of pre-warmed (37°C) RPMI complete in a 15 mL conical tube.
  c. Centrifuge at 400 × *g* for 5 min at 4°C.
  d. Resuspend cells in 10 mL of pre-warmed (37°C) RPMI complete and transfer to a 75cm^2^ tissue culture-treated flask.
  e. Incubate at 37°C and 5% CO_2_.

**CRITICAL:** The tumor cells will need to be able to grow in RPMI complete with M-CSF in order to be cultured with the macrophages during the assays. Some cell lines may do better with being slowly adjusted to RPMI complete in culture before the assay. Both adherent and non-adherent cells are compatible with the protocol. Non-adherent cells should be centrifuged to the bottom of the plate to facilitate rapid contact with macrophages.

## Key resources table

**Table:** 

REAGENT or RESOURCE	SOURCE	IDENTIFIER
**Antibodies**		

Brilliant Violet 711 anti-mouse CD45 (1:250 dilution)	BioLegend	Cat# 103147
Brilliant Violet 785 anti-human CD45 (1: 100 dilution)	BioLegend	Cat# 304048

**Chemicals, peptides, and recombinant proteins**		

0.25% trypsin EDTA	Thermo Fisher	Cat# 25200114
RPMI 1640 Medium	Thermo Fisher	Cat# 11875135
Penicillin-Streptomycin (10,000 U/mL)	Thermo Fisher	Cat# 15140122
GlutaMAX Supplement	Thermo Fisher	Cat# 35050061
Sodium pyruvate (100 mM)	Thermo Fisher	Cat# 11360070
MEM Non-Essential Amino Acids Solution (100×)	Thermo Fisher	Cat# 11140050
PBS, pH 7.4	Thermo Fisher	Cat# 10010049
HBSS, calcium, magnesium	Thermo Fisher	Cat# 14025126
Ficoll-Paque PLUS Media	Fisher Scientific	Cat# 45001749
Trypan Blue Solution, 0.4%	Thermo Fisher	Cat# 15250061
Recombinant Human M-CSF	PeproTech	Cat# 300-25
Recombinant Murine M-CSF	PeproTech	Cat# 315-02
Recombinant Human Lymphotoxin α1/β2 Protein	R&D Systems	Cat# 8884-LY
Recombinant Mouse Lymphotoxin α1/β2 Protein	R&D Systems	Cat# 9968-LY
Recombinant Human IFN-γ	PeproTech	Cat# 300-02
Recombinant Murine IFN-γ	PeproTech	Cat# 315-05
CellTrace Violet Cell Proliferation Kit, for flow cytometry	Thermo Fisher	Cat# C34557
CellTrace CFSE Cell Proliferation Kit, for flow cytometry	Thermo Fisher	Cat# C34554
LCL-161	Selleck Chemicals	Cat# S7009
Formalin solution, neutral buffered, 10%	Millipore Sigma	Cat# HT501128-4L

**Experimental models: Cell lines**		

6694c2-zsGreen-NLS	Engineered from 6694c2 from Kerafast (Cat# EUP006-FP) by overexpressing nuclear-localizing zs-Green	In-house
MDA-MB-231	ATCC	Cat# HTB-26

**Experimental models: Organisms/strains**		

Mice: Male or female C57Bl6/J mice, aged 7–16 weeks, strain 000664	The Jackson Laboratory	Cat#000664; RRID; IMSR_JAX;0000664

**Software and algorithms**		

Prism 9.3.0	GraphPad Software	https://www.graphpad.com/scientific-software/prism/
FlowJo 10.8.1	BD Biosciences	https://www.flowjo.com/

**Other**		

15 mL conical centrifuge tubes	Fisher Scientific	Cat# 1495970C
50 mL conical centrifuge tubes	Fisher Scientific	Cat# 1495949A
Round-bottom polystyrene test tubes	Fisher Scientific	Cat# 149595
12-well tissue culture-treated plates	Fisher Scientific	Cat# 07-200-82
150 × 15 mm Petri dishes	Fisher Scientific	Cat# 08-757-148
Cell strainer 40 μM	Fisher Scientific	Cat# 087711
27-gauge needle	BD	Cat# 305109
1 mL slip tip sterile syringes	Fisher Scientific	Cat# 14-823-434
Dissecting scissors	VWR	Cat# 82027-588
Dissecting forceps	VWR	Cat# 82027-406
Hemocytometer counting chamber	Fisher Scientific	Cat# 0267110

## Materials and equipment

**Table:** 

RPMI complete	Final concentration	Amount
RPMI 1640	N/A	860 mL
Penicillin-streptomycin (100×)	1×	10 mL
Glutamax (100×)	1×	10 mL
Sodium pyruvate (100×)	1×	10 mL
MEM NEAA (100×)	1×	10 mL
Inactivated fetal bovine serum	10%	100 mL
**Total**	**N/A**	**1 L**

Store at 4°C for up to 1 month.

**Table:** 

ACK lysis buffer	Final concentration	Amount
NH4Cl (Ammonium Chloride)	0.15 M	8.26 g
KHCO3 (Potassium bicarbonate)	0.01 M	1 g
EDTA	0.0001 M	0.037 mg
ddH_2_O	N/A	1 L
**Total**	**N/A**	**1 L**

Sterile-filter and store at RT.

**Table:** 

FACS buffer	Final concentration	Amount
PBS	N/A	488 mL
Inactivated fetal bovine serum	2%	10 mL
EDTA (0.5 M, pH 8.0)	2 mM	2 mL
**Total**	**N/A**	**500 mL**

Prepare prior to use.

## Step-by-step method details

### Macrophages replating procedure

**Timing: 1 day**

Before beginning the assay, macrophages must be reseeded into 12-well plates and given at least 8 h to attach. It is important at this stage to ensure that equal numbers of macrophages are seeded into each well. Macrophages can be replated after 5–6 days of culture and used for phagocytosis assessment at 7–12 days of culture, counting the initial removal from mouse femurs as Day 0. The following steps are applicable to both human and mouse macrophages unless otherwise stated.

1. Collect macrophages from petri dishes.
  a. Aspirate supernatant.
  b. Wash adherent macrophages with PBS.
    i. Prop petri dish at a 45° angle and rinse the plate from the top with 5–10 mL PBS. Aspirate PBS. If collecting mouse macrophages, gently rinse the plate once with PBS. If collecting human macrophages, rinse the plate more forcefully three times with PBS, as human macrophages are more difficult to remove from the plate.
  c. Dissociate macrophages.
    i. Add 5 mL of trypsin per plate and incubate up to 15 min at 37°C until cells are visibly detached from the plate.
  d. Collect macrophages.
    i. Prop petri dish up at a 45° angle and collect the trypsin into a 50 mL conical tube.
    ii. Using a 10 mL serological pipette filled with PBS, rinse plate from the top three times to collect remaining cells.
    iii. Collect all cells into the same 50 mL conical tube.
    iv. Centrifuge at 400 × *g* for 5 min at 4°C.
2. Prepare macrophages for plating.
  a. Count macrophages using a hemocytometer.
  b. Resuspend macrophages in RPMI complete supplemented with appropriate M-CSF depending on the species.
    i. Pipet 1 mL into each well of the 12-well plates at 25,000–100,000 cells/mL.
    ii. Aim for 100,000 cells per well on the day of the assay.
      ***Note:*** The macrophages will proliferate slightly over time, so if you do not plan on using the plates immediately, seed fewer than 100,000 cells per well.
  c. After plating macrophages, gently rock the plate from front to back and side to side to distribute the macrophages evenly.
3. Incubate at 37°C with 5% CO_2_ for at least 24 h.
  a. If not performing the phagocytosis assay within 2 days, give the macrophages additional RPMI complete with M-CSF every two to three days of culture.

### Steps for setting up phagocytosis assay

**Timing: 1 day**

On the day of the assay, tumor cells are washed and dissociated from their flask and plated onto macrophages. If the tumor cells are not fluorescent, then additional steps are needed to label the tumor cells before plating. Different treatments may be added prior to or during macrophage and tumor cell coculture to assess their impact on phagocytosis. Here, we include the SMAC mimetic LCL-161 and cytokines IFN-γ or lymphotoxin as agents known to induce phagocytosis of live tumor cells.^1^^,^^2^ The tumor cells should be as healthy as possible before being plated onto the macrophages, so keep the cells on ice during any waiting periods and try to move quickly.

4. Collect tumor cells (∗optional for non-adherent cells, collect cells and start from step e).
  a. Aspirate cell medium.
  b. Wash tumor cell flask three times with 10 mL of PBS.
  c. Dissociate tumor cells by adding 1 mL of trypsin 0.25% EDTA and incubating the flask at 37°C for 2–3 min.
  d. Quench the trypsin with 9 mL of RPMI complete and collect tumor cells into a 15 mL conical tube.
  e. Centrifuge at 400 × *g* for 5 min at 4°C.
  f. Aspirate supernatant.
  g. Resuspend tumor cells in 10 mL of HBSS.
  h. Centrifuge at 400 × *g* for 5 min at 4°C.
  i. Repeat steps f-h for two additional washes. During the second wash, take a small aliquot of the resuspended cells and count using a hemocytometer.

***Optional:*** If the tumor cells do not have a fluorescent marker, stain the cells with CFSE or other dye after counting. We typically use CellTrace CFSE or CellTrace Violet (CTV) for staining. Prepare CFSE or CTV as per the manufacturer’s protocol. Dilute CFSE/CTV in HBSS to a final concentration of 2 μM for CFSE or 5 μM for CTV. Pre-warm this HBSS-CFSE/CTV solution in the 37°C water bath until ready for staining. After three washes of the tumor cells with HBSS, resuspend the tumor cells at 1 million cells/mL in the HBSS-CFSE/CTV solution and place the cells in the 37°C water bath for 5 min (CFSE) or 20 min (CTV). Centrifuge at 400 × *g* for 5 min at 4°C and aspirate the HBSS-CFSE/CTV. Wash the cells three more times in ice-cold HBSS.

5. Prepare cells for plating.
  ***Note:*** The optimal tumor cell to macrophage ratio per well is 1:1, although other ratios may be used. If there are approximately 100,000 macrophages per well, prepare to plate at least 100,000 tumor cells per well – increase this tumor cell number based on the macrophage density. Do not exceed 500,000 cells total per well in a 12-well plate.
  a. Determine the resuspension volume for the tumor cells such that there is the desired number of tumor cells per well in a final volume of 1 mL per well. Resuspend cells in RPMI complete supplemented with the appropriate recombinant M-CSF.
  b. Keep the cells on ice until ready for plating.
    ***Note:*** If you are adding treatment during the coculture, make both the tumor cell resuspension and the treatment solution at 2× the final concentration, then add 500 μL of each to the wells. To make treatment solutions, first prepare RPMI complete with the species-specific M-CSF. Add in treatments at 2× the desired final concentration. These treatments can be prepared while the tumor cells are being washed.

6. Once the tumor cells and treatment media are ready for plating, aspirate the supernatant from the 12-well plates with reseeded macrophages.

***Optional:*** If the macrophages are pretreated, wash the 12-well plates three times with PBS before adding tumor cells. In order to avoid detachment of the macrophages, tilt the plate at a 45° angle to gently aspirate the supernatant from the side of the well and slowly pipet 1 mL of PBS onto the side of each well.

7. Plate tumor cells onto the macrophages.
  a. Prepare three replicate wells for each condition tested.
  b. If there is no treatment in the coculture, add 1 mL of the 1× tumor cell solution into each well of macrophages.
  c. If there is treatment in the coculture, first add 500 μL of the 2× tumor cell solution and then add 500 μL of the 2× treatment solution into each well.
  d. Remember to include a macrophage-only well and a tumor cell-only well as negative controls to help set the flow cytometry gates.
  e. After plating, gently rock the plate from front to back and side to side to distribute the cells evenly.
8. Incubate at 37°C with 5% CO_2_ for 18–24 h.

### Phagocytosis assay harvest and analysis

**Timing: 2 h**

After 18–24 h of coculture, the cells are ready to be collected, stained, and fixed for flow cytometry. The cells are dissociated from each well, collected into flow tubes, and stained for CD45 to identify the macrophages. The samples are then fixed and can be processed on a flow cytometer. All of the following steps can be performed on the benchtop.

9. Prepare flow cytometry tubes with 2 mL of HBSS for each well of the assay.
10. Pipet the 1 mL of supernatant from each well into the corresponding tube. This collects any non-adherent cells.
11. Add 1 mL of trypsin 0.25% EDTA into each well.
12. Incubate plates for 15 min at 37°C.
13. Collect the trypsinized cells into the corresponding flow tubes.
  a. The plates and tubes should be kept on ice during this process.
  b. Hold the plate at a 45° angle. Try to aim the pipette at different spots around the circumference of the well while pipetting up and down. The same tip can be used for wells of the same condition (troubleshooting 1).
  c. Check the plates under the microscope to ensure full collection of cells.
14. Centrifuge at 400 × *g* for 5 min at 4°C.
15. Prepare flow stain.
  a. For human cells, prepare 100 μL of FACS buffer with 1 μL of anti-human CD45 antibody for each sample with fewer than 0.5 million cells. For mouse cells, prepare 50 μL of FACS buffer with 0.2 μL of anti-mouse CD45 antibody for each sample with fewer than 0.5 million cells.
16. Decant the supernatant from the flow tubes without disturbing the pellets.
17. Add flow stain to the appropriate tubes and briefly vortex the tubes.
18. Stain for 15 min on ice, covered from light.
19. Add 200 μL of 1% formalin to each tube to fix.
20. Keep tubes at 4°C, covered from light, until ready to analyze on a flow cytometer (troubleshooting 2 and 3).

## Expected outcomes

**Figure 1:**
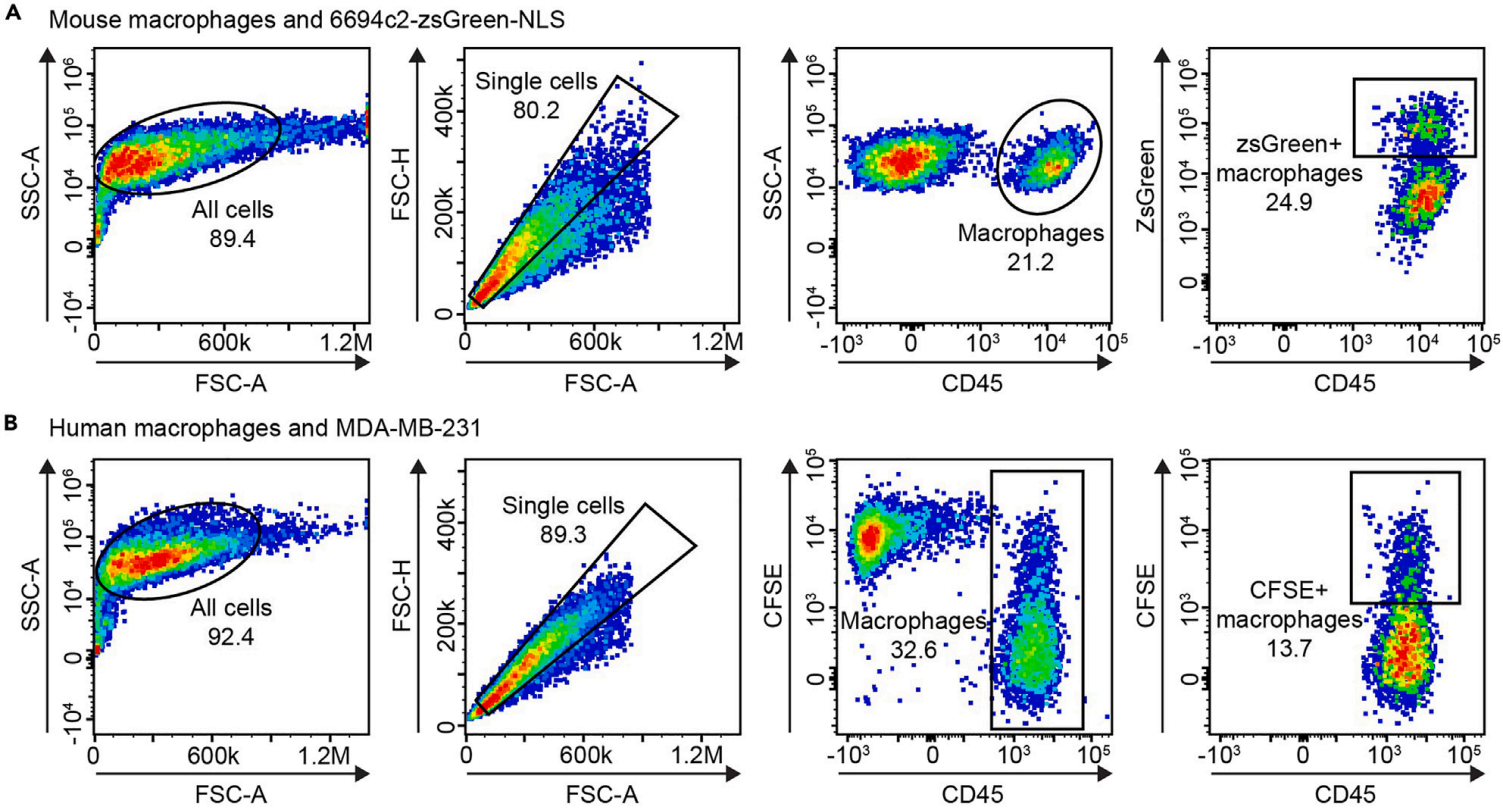
Gating strategy for phagocytosis assays (A and B) Mouse macrophages and 6694c2-zsGreen-NLS cells (A) or human macrophages and CFSE-stained MDA-MB-231 cells (B) were cultured together for 18 h. Phagocytosis was defined by flow cytometry, and the gating schemes are shown. FSC, forward scatter; SSC, side scatter; A, area; H, height.

**Figure 2:**
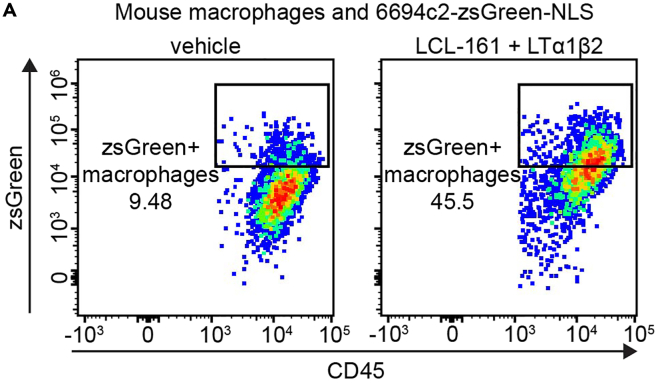
Representative plots showing LCL-161-induced increase in phagocytosis (A) Mouse macrophages and 6694c2-zsGreen-NLS cells were cocultured with vehicle or with 500 nM LCL161 and 10 ng/mL LTα1β2. Phagocytosis rates were determined by flow cytometry after 18 h of coculture. Flow plots are gated on CD45^+^ macrophages.

**Figure 3:**
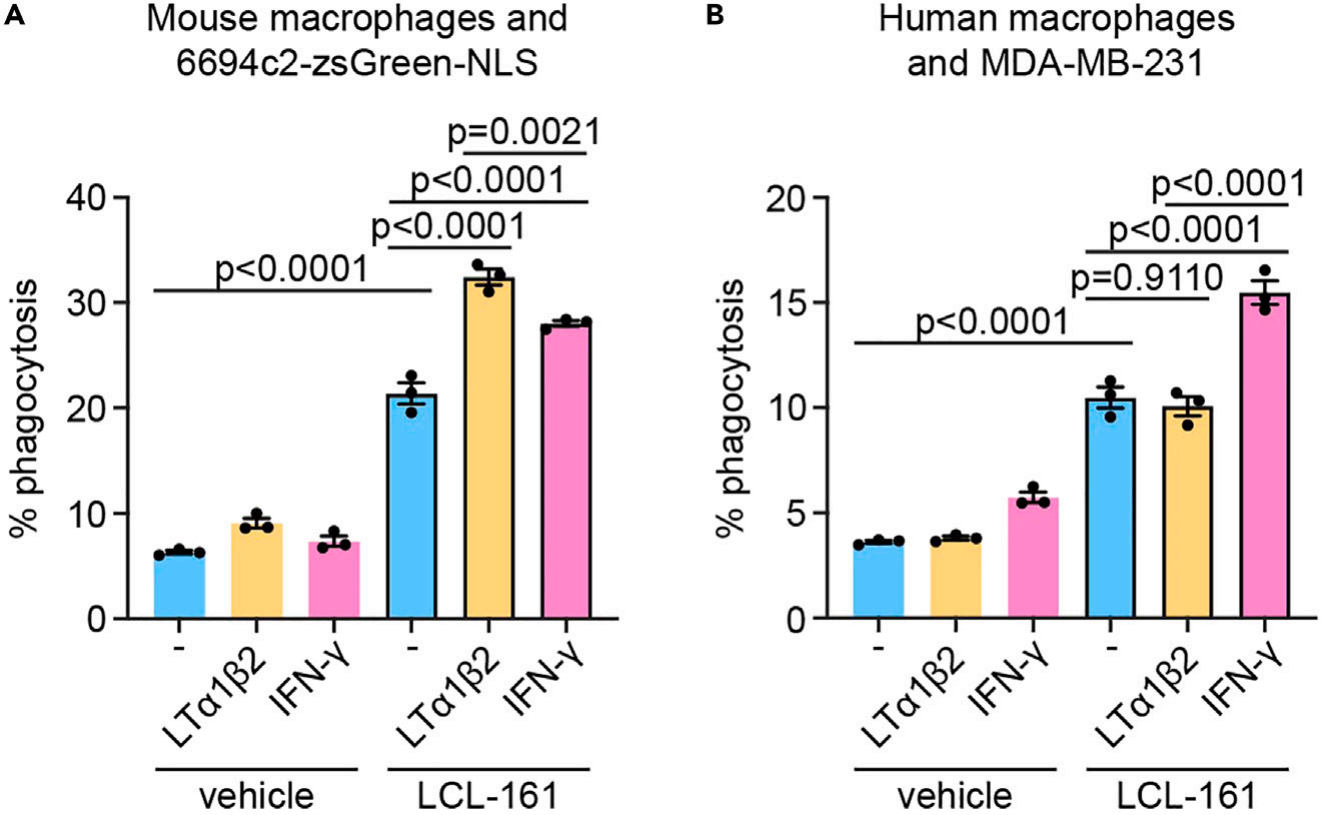
LCL-161 synergizes better with LTα1β2 in mouse macrophages but better with IFN-γ in human macrophages (A and B) Mouse macrophages and 6694c2-zsGreen-NLS cells (A) or human macrophages and CTV-stained MDA-MB-231 cells (B) were cultured together for 18 h with 500 nM LCL161 and LTα1β2 (10 ng/mL for mouse, 50 ng/mL for human) or IFN-γ (20 ng/mL for mouse, 50 ng/mL for human), or their controls. Data are representative of three independent experiments with three replicates per group. Data are presented as mean ± SEM and p-values were calculated by ANOVA.

A successful phagocytosis assay should show two distinct cell populations. One population of macrophages (CD45^+^) and one population of tumor cells (CD45^-^, positive for tumor cell marker, such as CFSE). The percentage of CD45+ cells that are also positive for the tumor cell marker indicate the macrophages that have taken up tumor cells (Figure 1). Here, we confirm that culturing macrophages with LCL-161 and IFN-γ or lymphotoxin induces phagocytosis of tumor cells^1^ (Figure 2). Although both cytokines can combine with LCL-161 to induce phagocytosis, lymphotoxin is better at inducing phagocytosis in mouse macrophages whereas IFN-γ is better at inducing phagocytosis in human macrophages (Figure 3).

## Limitations

While this assay gives a quantitative measure of tumor cell uptake by macrophages, it is not possible to distinguish whether the macrophages are engulfing live tumor cells or if they are taking up remnants from dead cells.^3^ We used video microscopy to show that LCL and lymphotoxin-treated macrophages do engulf live tumor cells,^1^ but this cannot be directly concluded from a flow-cytometry based phagocytosis assay. Therefore, it is important to keep the tumor cells healthy when plating onto macrophages and minimize the number of dead tumor cells present in the coculture. One possible solution is to stain the tumor cells with a viability dye prior to adding them to macrophages, but we have found this to compromise viability of the tumor cells, and it does not account for cell death that occurs after the start of coculture. Finally, the ratio of tumor cells to macrophages impacts the phagocytosis rate. If the tumor to macrophage ratio is low in some conditions, this might artificially inflate the measured phagocytosis rate. As such, it is important to keep the tumor cell to macrophage ratio consistent between conditions.

## Troubleshooting

### Problem 1

Incomplete collection of cells from the 12-well plates (related to step 13b).

### Potential solution

Macrophages adhere strongly to tissue culture-treated plates. Allow the plates to incubate with trypsin for 10–15 min at 37°C and pipet vigorously at points all around the circumference of the well to lift cells. If there are still cells attached, repeat the pipetting process with 1 mL of HBSS in each well to collect the remaining cells. We do not recommend using a cell scraper as this can damage the macrophages. Longer incubations up to 30 min with trypsin diluted in HBSS (no calcium or magnesium and 0.025% trypsin) or temperature-sensitive plates may be used to facilitate macrophage detachment.

### Problem 2

High measured phagocytosis rate across all conditions (related to step 20).

### Potential solution

It is critical to maintain the health of the tumor cells that are added to the macrophages. Unhealthy or dead tumor cells will lead to high rates of efferocytosis, making all CD45^+^ cells also positive for the tumor cell marker. Keep the tumor cells on ice throughout handling and optimize the staining procedure to minimize toxicity. Ensure that tumor cells are thoroughly washed before plating onto macrophages so that no residual CFSE or other stain is carried to the coculture.

### Problem 3

No phagocytosis is observed (related to step 20).

### Potential solution

This could be dependent on the cell line, as we have found that phagocytosis rates differ among different tumor cell lines. The baseline rate of phagocytosis of live tumor cells by macrophages should be low.^4^^,^^5^ Inclusion of LCL-161 or other positive control is critical to verify the technical success of the procedure. Tumor-specific antibodies that engage Fc receptors on macrophages should also induce phagocytosis,^6^^,^^7^ particularly when combined with CD47 blocking antibodies or the use of CD47−/− tumor cell lines.^8^^,^^9^^,^^10^^,^^11^

## Resource availability

### Lead contact

Further information and requests for resources and reagents should be directed to and will be fulfilled by the lead contact, Li Qiang (Li_Qiang@dfci.harvard.edu).

### Materials availability

This cell line 6694c2-zsGreen-NLS is available upon request.

## Data Availability

This study did not generate any datasets or code.
